# Back pain in children surveyed with weekly text messages - a 2.5 year prospective school cohort study

**DOI:** 10.1186/s12998-014-0035-6

**Published:** 2014-11-18

**Authors:** Claudia Franz, Niels Wedderkopp, Eva Jespersen, Christina T Rexen, Charlotte Leboeuf-Yde

**Affiliations:** Research in Childhood Health, Department of Sports Science and Clinical Biomechanics, University of Southern Denmark, Campusvej 55, 5230 Odense, Denmark; The Sport Medicine Clinic, Orthopaedic Department, Hospital of Lillebaelt, Lillebaelt, Denmark; Research Department, Spine Center of Southern Denmark, Hospital Lillebaelt, Middelfart and Institute of Regional Health Services Research, University of Southern Denmark, ᅟ, Denmark

**Keywords:** Children, Back pain, Text messages

## Abstract

**Background:**

Back pain is reported to occur already in childhood, but its development at that age is not well understood. The aims of this study were to describe BP in children aged 6–12 years, and to investigate any sex and age differences.

**Methods:**

Data on back pain (defined as pain in the neck, mid back and/or lower back) were collected once a week from parents replying to automated text-messages over 2.5 school years from 2008 till 2011. The prevalence estimates were presented as percentages and 95% confidence intervals. Differences between estimates were considered significant if confidence intervals did not overlap. A test for trend, using a multi-level mixed-effects logistic regression extended to the longitudinal and multilevel setting, was performed to see whether back pain reporting increased with age.

**Results:**

Depending on the age group, 13-38% children reported back pain at least once per survey year, and 5-23% at least twice per survey year. The average weekly prevalence estimate ranged between 1% and 5%. In the final survey year more girls than boys reported back pain at least twice. The prevalence estimates did not increase monotonically with age but showed a greater increase in children younger than 9/10, after which they remained relatively stable up to the age of 12 years.

**Conclusions:**

We found that back pain was not a common problem in this age group and recommend health professionals be vigilant if a child presents with constant or recurring back pain. Our results need to be supplemented by a better understanding of the severity and consequences of back pain in childhood. It would be productive to study the circumstances surrounding the appearance of back pain in childhood, as well as, how various bio-psycho-social factors affect its onset and later recurrence. Knowledge about the causes of back pain in childhood might allow early prevention.

**Electronic supplementary material:**

The online version of this article (doi:10.1186/s12998-014-0035-6) contains supplementary material, which is available to authorized users.

## Background

Back pain (BP) is reported already in early childhood [1,2] and at least low back pain (LBP) accelerates in puberty [1,3,4]. However, little is known about the time of onset in childhood and the subsequent course of BP.

Epidemiologic studies of BP seldom include younger children and results are typically reported for age groups rather than for each year separately. Children are not easy to survey. They may find it difficult to answer a questionnaire due to insufficient language skills and problems in relating to pain and how to grade it, and also because of their limited understanding of the concept of time. It has, for example, been shown that children often have a limited memory of past or recurrent “ordinary” events, and can more easily remember unique and distinctive experiences [5]. As surveys on BP usually deal with recall periods beyond “today”, this is a challenge, particularly when questions are asked about pain during the preceding year, a recall period often used in BP research.

Most studies on BP in the younger population thus concentrate on older children [6,7]. The paucity of valid data in younger children makes it difficult to determine the age at which BP starts to occur.

New research tools allow frequent data collection at low cost, thus removing much of the recall problem and enabling larger study samples that can distinguish between age groups in more detail and over longer periods of time.

The purpose of this study was to generate descriptive information on BP in children aged 6 to 10 years, who were surveyed weekly over 2.5 school years with automated text messages. We sought to obtain answers to the following questions:

1. What is the proportion of children reporting BP?
  i. at least once in a school year?
  ii. at least twice in a school year?
2. What is the average weekly proportion of children reporting BP during a school year?
3. Is there a difference in BP reporting between girls and boys?
4. Does BP reporting increase with age?

We expected the prevalence of BP to be fairly low, but that it would increase gradually with age or that there might be a cut-point when it would increase markedly. We also expected the vast majority of children who reported BP to do so only once, and that girls would have a higher prevalence of BP than boys.

## Methods

### Design

Longitudinal data from the Childhood Health, Activity and Motor Performance School Study Denmark (CHAMPS Study-DK) collected between October 2008 and July 2011 were used [8]. The CHAMPS study was a large prospective school-based project in the form of a natural experiment [9], which evaluated the effect of increased physical education on childhood health in general. The study was undertaken in Svendborg, Denmark, a municipality situated in a rural area with 59,000 inhabitants. The method of this study has been extensively described elsewhere [8]. The present study used only the CHAMPS data on BP (defined as pain in the neck, mid back and/or lower back) that were collected weekly with automated text messages.

### Study population

The CHAMPS study included children in pre-school (grade 0) up to fourth grade in ten public schools. All the children also agreed to participate in the weekly registration of BP using automated mobile phone text messages. To allow for a phasing-in process, schools were included gradually between November 2008 and August 2009. The study was kept open, with the possibility for new children to enter.

The text message data were collected over 2.5 school years (“survey years”). Thus, in the first survey year (2008/9), the children were in grades 0–4. In the second survey year (2009/10), these children were in grades 1–5 and in the third survey year (2010/11), they were in grades 2–6 (Table 1).

**Table 1:** **The distribution of school grades in the subsequent survey years**

**First survey year** →	**Second survey year** →	**Third survey year** →
Grade 0	Grade 1	Grade 2
Grade 1	Grade 2	Grade 3
Grade 2	Grade 3	Grade 4
Grade 3	Grade 4	Grade 5
Grade 4	Grade 5	Grade 6

As Danish children rarely repeat their first school years, grade 0 pupils are typically 6/7 years old, grade 1 children are 7/8 years, grade 2 children are 8/9 years, grade 3 children are 9/10 years and grade 4 children are 10/11 years old. School-grade was thus considered a proxy for age.

The study results can be viewed in two different ways: i) The estimates of BP for each grade can be interpreted in relation to the other grades for each survey year (i.e. comparing different children) or ii) the estimates of BP can be followed longitudinally over the 2.5 survey years (i.e. following the same children over time).

### Data collection from parents

As part of the CHAMPS study, weekly information on BP was collected using automated text messages (SMS-Track) each week from November 2008 until June 2011, except during the six weeks of summer holiday [8]. Every week on Sunday, the parents received the following question: “Has [NAME OF CHILD] during the last week had any pain in: 1. Neck, mid back and/or lower back, 2. Shoulder, arm or hand, 3. Hip, leg or foot and 4. No my child has not had any pain. The parents were asked to type the number in front of the correct answer in a return text message. Data used in this report related to items 1 and 4. Also information from a detailed questionnaire on the health of the child was available, as parents had filled in a questionnaire at baseline [8].

### Quality of the SMS-Track data

The returned answers were automatically recorded and inserted into a database. A reminder was sent automatically if a response had not been received within 72 hours and, if necessary, again 120 hours after the initial text message was sent. The SMS-Track data were monitored and cleaned during data collection, and any inappropriate answers (e.g. a response in words) were checked through direct telephone contact with the parents.

The information was collected from parents to ensure continuity in data collection over several years. Proxy reports of children’s BP were considered appropriate in this cohort, as self-report questionnaires in young children might be inaccurate [10-13]. A validation study was undertaken in order to determine the reproducibility of the SMS-Track reporting when comparing it with verbal reporting. The sensitivity for the SMS data was 0.98, specificity 0.87, positive predictive value 0.94 and the negative predictive value 0.95, indicating high validity of data [14].

### Clinician-generated data

Parents who reported that their child had pain in the previous week were contacted by telephone at the beginning of the subsequent week by one of four clinicians. During the contact the specific location of pain (neck, mid back and/or lower back) and pain history were systematically recorded. If symptoms still persisted, the child was examined by a chiropractor, physiotherapist or a medical practitioner within the next fortnight.

Injuries were diagnosed according to the International Classification of Diseases (ICD-10) [15]. If necessary the child was referred for further para-clinical examination, such as X-ray, ultrasound or magnetic resonance imaging scan. If pathology was found the child was referred to relevant medical specialists for further examination and treatment. (Data on clinician-generated data and diagnosed back pain to be reported elsewhere).

### Ethical approval

Ethics committee approval was obtained for the CHAMPS study (ID S20080047) and the study was registered with the Danish Data Protection Agency, as stipulated by the law J.nr. 2008-41-2240. Written informed consent was obtained from parents. Every parent and child also gave verbal acceptance prior to every clinical examination. All participation was voluntary with the option to withdraw at any time.

### Data analysis

STATA 11.0 (StataCorp, College Station,Texas, USA) was used for data analyses. Some faulty answers were provided during the start-up-phase at each school, probably because of the novelty of the method. In the beginning it was therefore necessary to contact some of the parents in order to re-explain the correct use of the SMS-Track method. We thus considered the first 9 weeks of data collection at each school to be a pilot phase and data from that period were completely excluded from analysis. The resulting data for analysis were thus collected over 22 weeks in the 1^st^ survey year, 43 weeks in the 2^nd^ survey year and 44 weeks in the 3^rd^ survey year, giving a total of 109 weeks.

Prevalence estimates of BP at least once a survey year were based only on data from the 2^nd^ and 3^rd^ survey years (as the first survey year did not cover an entire school year). However, analysis of average weekly BP prevalence included data also from the first survey year.

BP reporting was determined for each grade in the survey years, first in relation to at least one BP report per individual and then for the number of BP reports per individual. All analyses were stratified by sex, but where there were no clear differences, results are reported for girls and boys together. Estimates were calculated using one decimal figure but are reported to the nearest whole figure, where 0.5 was rounded up. Differences between estimates were considered significantly different, if their 95% confidence intervals (CI) did not overlap.

Initially, weighted estimates were calculated to give more influence to the text messages from those parents who were consistently compliant, compared to those who only answered occasionally. The high response rate meant that the weighted and unweighted estimates were almost identical, however, and thus weighting of the data was abandoned.

A test for trend, using a multi-level mixed-effects logistic regression extended to the longitudinal and multilevel setting, was performed to see whether BP reporting was positively associated with age. Classes were grouped into three classes per survey year. In the first survey year, grade 0 was considered the first “class”, grade 1 the second “class” and grades 2–4 the third “class”. In the second survey year baseline grade 0, now grade 1, was defined as the first “class”, grade 2 the second “class” and grades 3–5 the third “class”. In the last survey year grade 2 was defined as the first “class”, grade 3 the second “class” and grades 4–6 the third “class”. Children, classes and schools were random effects and the explanatory variables were sex and the three classes. Potential patterns of missing values were analyzed using logistic regression analysis. Missing values because of practicalities concerning changed or wrong mobile numbers were dropped for analyses.

## Results

### Participants and text messages

Overall participation in the CHAMPS study was 1,218 children (81%) from ten schools. There were 113 dropouts due to children moving away from the municipality or changing to non-project schools. These dropouts were counterbalanced by 121 new children moving to project schools, due to normal demographic mobility [8]. Fifteen children dropped out for other reasons, mainly because answering text messages every week was considered too bothersome. Data from these dropouts were included in the analysis for as long as they participated in the study.

In principle all children were included in the study. However, four children had to be excluded from analysis as they had serious musculoskeletal pathologies at baseline. Thus there were 765, 1164 and 1171 children in the data analyses for the three survey years (Table 2). There were slightly more girls than boys in each survey year.

**Table 2:** **Number of participants and percentage of females in each grade and survey year**

**School grade in survey year 1**	**Participants in survey year 1**		**Participants in survey year 2**		**Participants in survey year 3**	
	**N**	**% girls**	**N**	**% girls**	**N**	**% girls**
**0**	133	56%	207	53%	206	55%
**1**	160	55%	236	55%	237	54%
**2**	157	46%	251	46%	259	46%
**3**	155	51%	233	55%	233	55%
**4**	160	58%	237	62%	236	55%
**Total/Mean**	765	53%	1164	53%	1171	53%

The average weekly response rate for SMS Track was 96.5% over the 109 weeks with a total of 108,283 observations recorded altogether. No pattern for missing values was found, thus these values were excluded from the analyses.

### Proportion of children reporting BP at least once in a survey year

**Figure 1:**
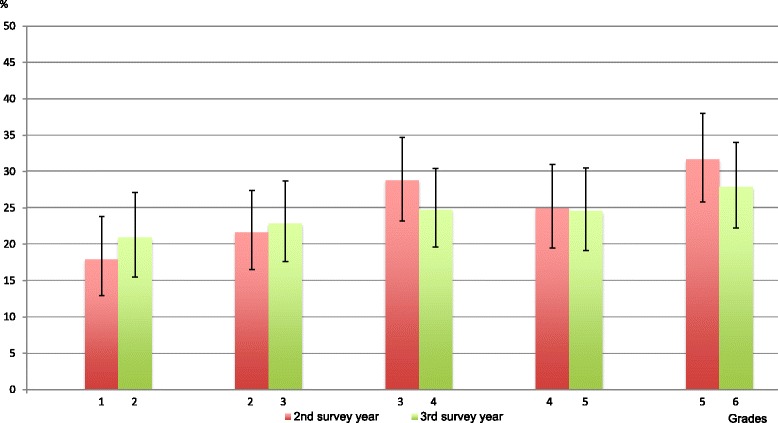
**Percentage of children in each grade with BP at least once in a school year.**

During the second or third survey years, three-quarters of children never reported any BP. The overall prevalence of BP was 25% [95% CI 23–28] in the second survey year for children in grades 1–5 and 24% [95% CI 22–27] in survey year three for children in grades 2–6. Prevalence of “BP at least once” was thus similar from one survey year to the next. Results on grade level ranged between 13% and 38%, when taking their confidence intervals into consideration (Figure 1).

No differences were seen in BP prevalence between girls and boys. However, as seen in Figure 1, BP became more common with age. This was confirmed with the test for trend on the data from the second survey year for grades 1–3, whereas the trend was not recognizable after third grade. Thus BP estimates increased from grades 0–3 and remained relatively stable after grade 3. Similar but less distinct findings were noted in the third survey year, when children were older.

### Proportion of children reporting BP at least twice in a survey year

**Figure 2:**
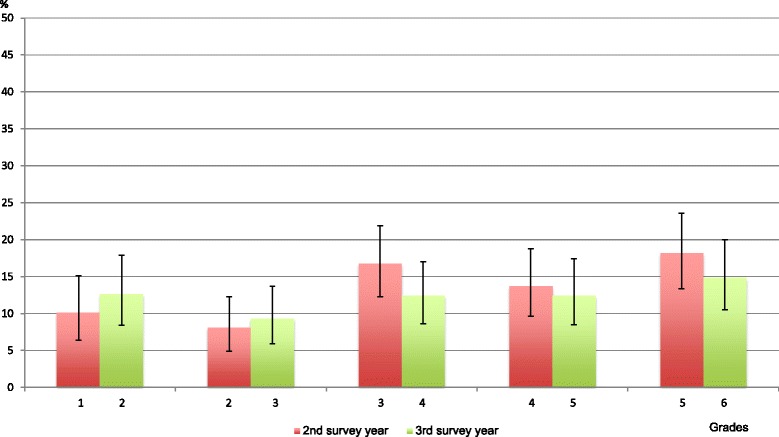
**Percentage of children in each grade with BP at least twice in a school year.**

Overall mean prevalence of BP at least twice was 13% [95% CI 11.5-15.5] in the second survey year and 12% [95% CI 10.5-14] in survey year three. Overall prevalence rates were thus similar in the two survey years and were almost half the prevalence of BP reported at least once. Results on grade level ranged between 5% and 23%, when taking their confidence intervals into consideration (Figure 2).

**Figure 3:**
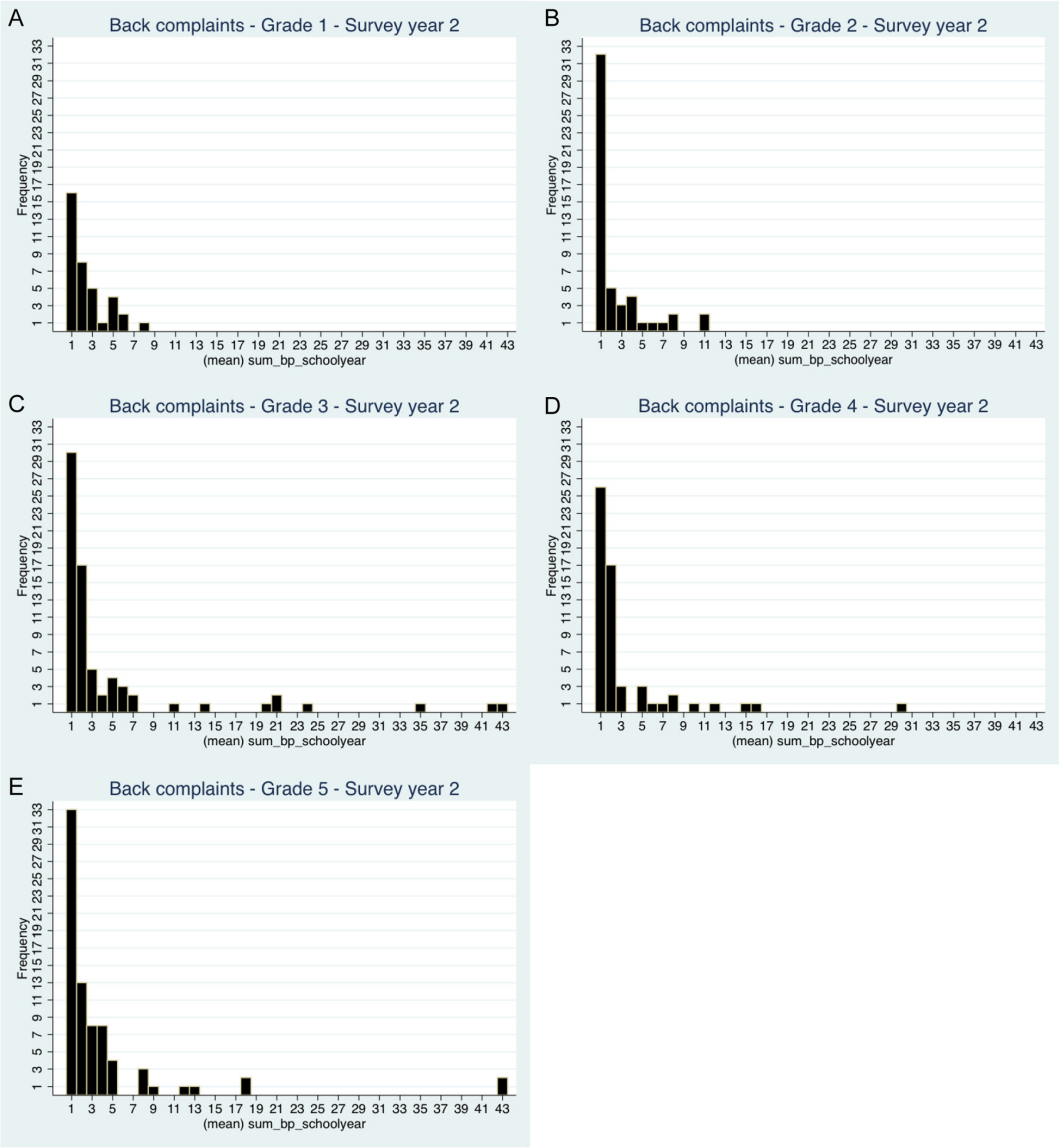
**a-e. Frequency of reported BP in each school grade, survey year two.**

Prevalence of BP at least twice was similar for girls and boys except in the 3^rd^ survey year, where the overall prevalence was 15% [95% CI 13–18.5] for girls and 9% [95% CI 6.5-11] for boys (Additional file 1). As seen in Figures 3a-e., it was not common to report BP more than once but the prevalence increased with age. The test for trend on the data from the second survey year showed increased BP estimates from grades 1–3 and again, relatively stable estimates after grade 3.

### Average weekly proportion of children reporting BP

Between 1% and 5% of children reported BP each week, with the lowest proportions in the lowest grades (Table 3).

**Table 3:** **Average weekly percentage of children with back pain in each grade and survey year**

**School grade in survey year 1**	**Average weekly % (n) of children with back pain**					
	**Survey year 1**		**Survey year 2**		**Survey year 3**	
**0**	1%	(n = 1)	1%	(n = 2)	2%	(n = 3)
**1**	1%	(n = 2)	1%	(n = 3)	1%	(n = 3)
**2**	5%	(n = 7)	3.5%	(n = 9)	3%	(n = 8)
**3**	3%	(n = 4)	2%	(n = 5)	2%	(n = 5)
**4**	3.5%	(n = 5)	3%	(n = 7)	3%	(n = 6)

Visual inspection revealed that estimates were 1-7% in girls and 1-6% in boys. The test for trend revealed increased BP estimates for grades 0–2 in the first survey year and for grades 1–3 in the second survey year, whereas the trend was not recognizable after grade 2 in the first survey year and grade 3 in the second survey year. Similar but less distinct findings were noted in the third survey year, when children were older.

## Discussion

This is the first study assessing back pain in age-specific cohorts in childhood, where weekly follow-up was performed over a long period of time. We found that BP was relatively uncommon in childhood and occurred mainly as a single event, thus not as a recurring or chronic condition as often seen in adults. There was a tendency for the older age groups to have a wider spread in the number of times BP was reported. More girls than boys reported BP at least twice in the 3^rd^ survey year. An increase in BP reporting was seen in the first and second survey years, especially in children younger than 9/10 years and remained fairly stable up to the age of 12 years.

BP was fairly uncommon in this study group. In one full survey year, 75% of the 7–12 year-old children reported “no BP”. Also a previous study reported that 78% of 11–14 year-olds belonged to the “no BP problem” cluster [16], where BP was defined as “pain in the past three months that lasted a whole day or more, or that had occurred several times in a year” and follow-up was every 3^rd^ month for 3 years.

### Comparison to other studies – BP at least once

We identified one comparable article in which BP was reported as at least once over a certain recall period of some of the relevant age group (Table 4). As the recall period in that study was only one month it would be expected that the prevalence estimates were lower than ours. However, 33% of 9 year-olds and 28% of 13 year-olds reported BP [7]. This compared to 25% in our 9/10 year olds and 28% of those aged 12/13.

**Table 4:** **Data from the epidemiologic literature on back pain at least once in children**

	**Mikkelsson et al.** **[** 17 **]**	**Hakala et al.** **[** 18 **]**	**Petersen et al.** **[** 19 **]**	**Stanford et al.** **[** 20 **]**	**Dunn et al.** **[** 16 **]**	**Kjær et al.** **[** 7 **]**
**Country**	Finland	Finland	Sweden	Canada	USA	Denmark
**Design**	Cross sectional + follow up	Cross sect. + follow up	Cross sectional	Longitudinal (8 yrs)	Longitudinal (3 m/3 yrs)	Cross sectional + follow up
**Study sample**	Pupils from 19 primary schools	Population register. All Finns born on adjacent dates in summer	Randomized cluster sample of pupils	Non-institutionalized civilian population	Girls/boys initially 11 yrs randomly selected in GH database	Primary/secondary school. 38 state schools in one municipality
**Response rate**	83%	77%	97%	?	49%	62%, 57%, 58%
**Valid sample size**	1756	62677	1121	2488	1333	479, 439, 443
**Data collection**	Questionnaire	Questionnaire	Questionnaire	Computer ass. Interview + Questionnaire	Telephone survey + Questionnaire	Interview + Questionnaire
**Age group**	9, 12 (mean: 9.8, 11.8)	12,14,16,18 (mean: 12.6, 14.6, 16.6, 18.6)	6-13	10-18	11-14	9,13,15 (mean: 9.7, 13.1, 15.7)
**Definition of back pain**	Pain/ache in neck, upper back (UB), low back (LBP)	Back or neck pain the past half a year	Backache last 6 months	Backache past 6 months	Back pain a whole day or more in the past 3 months	Any spinal pain:
**Recall period**	3 months	6 months	6 months	6 months	3 months	1 month
**Prevalence estimates never/seldom**	BP (UB, LBP)	BP + NP (12y)	BP (6-13y)	BP (12-13y)	BP (11-14y) 78%	BP (9y, 13y, 15y) 67%
**Overall: monthly**						33%, 28%, 48%
**Frequency**	Weekly	Weekly	≥ 1 Weekly:	Weekly	Low/high	
**Once**					10% (low prob.)	
**Several/frequently/continually**	12.7% (Only mentioned in the discussion section)	Girls: 6-16% Boys: 4-10%	Grade 0: 2% Grade 1: 3% Grade 2: 3% Grade 3: 6% Grade 4: 7.5% Grade 5: 14% Grade 6: 17%	17.6%	1,3% (high prob.)	

### Comparison to other studies – BP at least twice

As only six of the children in our study reported BP every week for a whole school year, it is unlikely that many children experience “pain every week”, a definition found in some previous research (Table 4).

We suspect that prevalence of BP as high as 14% and 17% in grades 5 and 6 [19] were artificially high due to using longer recall periods (e.g. 6 months). High estimates of pain every week for 6-16% of girls and 4-10% of boys aged 12 years [18] and of 18% in 12/13 year-old adolescents have also been reported [20].

### Comparison to other studies – average weekly pain reporting

Our estimates for BP would be expected to be higher than results from two studies that asked children only about LBP using a recall period of one week [21,22]. This was not the case, however, as a Japanese study found point estimates for LBP in 9–12 year-olds of 3-6% [22], and a British study reported point estimates of LBP of 6-11% for boys and 9-13% for girls aged 10–13 years [21]. This compared to 1-5% in our 9–12 year-olds, 1-5% for boys and 2-5% for girls aged 10–13 years. A Swiss study reported that 16% of schoolchildren had complained of BP the previous week. However, this was an overall estimate of BP for students aged 7–17 years [1] (Table 5).

**Table 5:** **Data from the epidemiologic literature on “one week” prevalence of back pain (including LBP region)**

	**Balague et al.** **[** 1 **]**	**Jones et al.** **[** 21 **]**	**Sato et al.** ** [** 22 **]**
**Country**	Switzerland	England	Japan
**Design**	Cross sectional	Cross sectional	Cross sectional
**Study sample**	Schoolchildren in primary and secondary school (one school district)	Schoolchildren in three school districts	Elementary and junior high schoolchildren in Niigata City
**Response rate**	99%	93%	79,8%
**Valid sample size**	1666	500	34423
**Data collection**	Questionnaire	Questionnaire	Questionnaire
**Age group**	7-17 (mean 12)	10-16	9-15
**Definition of back pain**	BP (=all spinal pain)	LBP	Any LBP now
**Recall period**	Previous week	Previous week	Now
**Overall - prevalence week/now**	BP (7-17y)	LBP	LBP
	16%	10-13 y: Boys, Girls	9-10 y: 3%
		10-11y: 6%, 9%	10-11 y: 4%
		11-12 y: 9%, 10,5%	11-12 y: 6%
		12-13 y: 11%, 13%	12-13 y: 12%
		13-14 y: 13%, 17%	13-14 y: 17%
		14-15 y: 18%, 21%	14-15 y: 15%
		15-16 y: 23%, 21%	

### Comparison to others - do girls report BP more often than boys?

Girls appear to be more likely to express distress in response to pain than boys [23] and to give higher ratings of pain than boys [24]. Differential socialization or specific hormonal and biochemical mechanisms may contribute to these sex differences [20]. This has been seen also in other studies [1,18,20,25,26] from the age of 13, where girls were more likely to report BP than boys (Tables 6 and 7). However, below this age some studies reported no sex differences [7,19]. Our estimates of BP in boys and girls were similar when BP at least once a survey year was analyzed, but in the last survey year, girls were more likely than boys to report BP at least twice.

**Table 6:** **Data from the epidemiologic literature on back pain in boys and girls (age included)**

	**Balague et al.** **[** 1 **]**	**Brattberg et al.** **[** 25 **]**	**Taimela et al.** **[** 6 **]**	**Hakala et al.** **[** 18 **]**	**Watson et al.** **[** 12 **]**
**Country**	Switzerland	Sweden	Finland	Finland	England
**Design**	Cross Sectional	Cross sectional + follow up	Cross sectional	Cross sect. + follow up	Cross sectional
**Study sample**	Schoolchildren in primary and secondary school- one school district	Pupils from 26 urban schools	Pupils from 45 different public schools	Population register. All Finns born on adjacent dates in summer (1985–9, 1993–7)	Pupils from secondary schools; state + private, urban + rural
**Response rate**	99%	87%	82%	77%	92% (LBP)
**Valid sample size**	1666	1245/ 471	1171	62677	1376 (LBP)
**Data collection**	Questionnaire	Questionnaire	Questionnaire	Questionnaire	Questionnaire
**Age group**	7-17(mean 12)	8, 11, 13, 17	7, 10, 14, 16	12,14,16,18(12.6, 14.6, 16.6, 18.6)	11-14
**Definition of back pain**	LBP, BP (=all spinal pain)	Do you often have back pain?	LBP interfering with school/leisure activities + recurrent LBP past 12 months	Back or neck pain during the past half a year	LBP for one day or longer in the past month
**Gender**	Girls > Boys (+BP ++LBP)	Girls > Boys all age groups. Significant among the 13 and 17-year-old pupils	No general difference. Girls > boys in recurrent LBP reporting	Girls > boys No interaction between sex but increasing trend was seen in girls – boys U shaped curve	Girls > Boys
**Age (prevalence increase)**	>13	Trend of more long-lasting BP in older age groups. Especially among girls	Recurrent LBP increases > 14, 16	Prevalence increased with age	Increase with age in girls and boys

**Table 7:** **Data from the epidemiologic literature on back pain in boys and girls (age included)**

	**Petersen et al.** ** [** 19 **]**	**Grøholt et al.** **[** 26 **]**	**Sato et al.** **[** 22 **]**	**Stanford et al.** **[** 20 **]**	**Kjær et al.** **[** 7 **]**
**Country**	Sweden	Nordic countries	Japan	Canada	Denmark
**Design**	Cross sectional	Cross sectional	Cross sectional	Longitudinal – 8 yrs	Cross sectional + follow up
**Study sample**	Randomized cluster sample of pupils	Population registries children survey	Elementary and junior high school-children in Niigata City	Non-institutionalized civilian population (1994–5, 1996–7, 1998–9, 2002–3)	Primary/secondary school. 38 state schools in one municipality
**Response rate**	97%	64.5-69%	79.8%	?	62%, 57%, 58%
**Valid sample size**	1121	5911 (BP)	34423	2488	479, 439, 443
**Data collection**	Questionnaire	Questionnaire	Questionnaire	Computer ass. Interview + Questionnaire	Interview + Questionnaire
**Age group**	6-13	7-9, 10–12, 13–15, 16-17	9-15	10-18	9, 13, 15 (mean 9.7, 13.1, 15.7)
**Definition of back pain**	Backache the last 6 months	Has the child had any of the following complaints? (BP, headache e.g.)	Any LBP now	Backache past 6 months	Any spinal pain
**Gender**	No gender difference	Girls > boys in all pain categories	11-12y girls > boys	Girls > boys	No difference in overall back (spinal) pain reporting at age 9 and 13 yrs.
**Age (prevalence increase)**	Prevalence of bachache higher from grades 4–6 than in grades 0–3 (Method change)	BP + headache most prevalent in the oldest age groups compared to the youngest	Increasing prevalence with grade levels until age 14 (LBP: Point prevalence)	Girls 12–18 yrs > boys 12–18 yrs	> 13 yrs

### Comparison to others – does BP increase in this age group?

Our results showed increased reporting of BP up to the age of 9/10 years, after which reporting appeared to be fairly stable. Previous studies have also reported a significant increase before the age of 12/13 years [19,22,26], but even more after this age [1,6,7,12,18,22,26] (Tables 6 and 7). It would be interesting to follow children into puberty, a time that has previously been identified as the period of acceleration of spinal pain (Tables 6 and 7). As there is no clear step-wise increase in BP reporting, it is unlikely that BP is caused only by the mere “burden of living”, i.e. it does not seem to be explained by the wear and tear of physical activities that accumulates over the childhood years.

### Methodological considerations

The major strengths of this study are that the study sample was taken from the real world in a natural experiment, and that the sample size was fairly substantial. Memory decay would be unlikely, as data were collected weekly. Bias in reporting was also unlikely, as there was an exceptionally high response rate (96.5%) and data were collected consistently over 2.5 school years, which provided a unique opportunity to follow these children very closely over time. Parental reports were used as proxy measurements for their children’s experiences of pain, which was both a potential strength and a weakness.

An earlier study [19] that used parental-assisted responses from children in grades 0–4 found rather low BP estimates, somewhat comparable to ours. However, in that study, when the children were aged 11/12 years, the data collection method changed so that the children completed the questionnaires themselves. At that time their prevalence estimates doubled, going from a weekly frequency of 7.5% to 14% [19]. It is uncertain which of the estimates (if any) was the most valid report. Parents are more likely to agree with their child on reporting LBP if disability levels are high [12] and on conditions that are common, visible, or diagnosed e.g. in longstanding illness [27]. However, we do not know how reliable the child–parent communication is on less severe pain and with frequent data collection. We hoped that the frequent text-message procedure would stimulate them to reflect and communicate appropriately. Asking children to report pain retrospectively over months or even a year will probably result in less valid answers and probably overestimation [3,28].

A potential weakness is that our study population lived in a medium-sized Danish rural municipality, which might have a different reporting pattern compared to a study population in larger cities or in other cultures. However, the comparison with the results in other studies reveals only logical differences, more related to the method of data collection than geographical or cultural differences. Other potential weaknesses were that we did not adjust for amount and type of physical activity. As half of the children received extra physical education lessons, this may have affected the estimates, although possibly in either direction. Other extrinsic or intrinsic factors will be taken into account in future studies. Furthermore, data gathered from the clinicians were not included in the manuscript. Also the latter topic will be dealt with in other reports.

## Conclusion

BP does not appear to be a major problem in childhood. Knowledge about the causes of BP in childhood might allow early prevention, however, and the topic is therefore important from a public health viewpoint.

It would be productive for further research to study the circumstances surrounding the appearance of back pain in childhood, as well as how various bio-psycho-social factors affect its onset and later recurrence. A better understanding of the severity and consequences of back pain in childhood is also needed.

From a clinical viewpoint, health professionals should be vigilant if children present with constant or recurring back pain, as such a pattern appears to be unusual in this population group.

## Additional file

Additional file 1: Table S1. Number of children with back pain at least once in a survey year. **Table S2.** Number of children with back pain at least twice in a survey year. **Table S3.** Number of girls with back pain at least twice in a survey year. **Table S4.** Number of boys with back pain at least twice in a survey year.
